# Antiviral and Immunomodulatory Activities of *Clinacanthus nutans* (Burm. f.) Lindau

**DOI:** 10.3390/ijms241310789

**Published:** 2023-06-28

**Authors:** Chung-Ming Lin, Hsin-Han Chen, Chi-Wen Lung, Hui-Jye Chen

**Affiliations:** 1 Department of Biotechnology, School of Health Technology, Ming Chuan University, Taoyuan 33348, Taiwan; lin.chungming@gmail.com; 2 Division of Plastic and Reconstructive Surgery, Department of Surgery, China Medical University Hospital, Taichung 40402, Taiwan; scapulachenhh@yahoo.com.tw; 3 Department of Creative Product Design, Asia University, Taichung 413305, Taiwan; cwlung@asia.edu.tw; 4 Graduate Institute of Biomedical Sciences, China Medical University, Taichung 40402, Taiwan

**Keywords:** *Clinacanthus nutans*, antiviral activity, immunomodulatory activity, phytochemicals

## Abstract

*Clinacanthus nutans* (Burm. f.) Lindau has been used as a traditional herbal medicine for treating snake bites, scalds, burns, and viral and bacterial infections. It has been attracting an increasing amount of attention because of its biological activities, including its antidiabetic, antioxidant, antibacterial, anticancer, anti-inflammatory, antiviral, and immunoregulatory activities. Here, we conducted a panoramic survey of the literature regarding the immunoregulatory, anti-inflammatory, and antiviral activities of *C. nutans*. We discovered that *C. nutans* extracts have virucidal activities against herpes simplex virus types 1 and 2, varicella-zoster virus, cyprinid herpesvirus 3, porcine reproductive and respiratory syndrome virus, mosquito-borne chikungunya virus, and potentially SARS-CoV-2; such activities likely result from *C. nutans* interfering with the entry, penetration, infection, and replication of viruses. We also reviewed the phytochemicals in *C. nutans* extracts that exhibit anti-inflammatory and immunoregulatory activities. This updated review of the antiviral, anti-inflammatory, and immunoregulatory activities of *C. nutans* may guide future agricultural practices and reveal clinical applications of *C. nutans*.

## 1. Introduction

*Clinacanthus nutans* (Burm. f.) Lindau is a medicinal herb that belongs to the *Acanthaceae* family, which contains many useful medicinal plants [1]. This plant is widely found in subtropical Asian countries, including Thailand, Indonesia, Malaysia, and China [1]. Traditionally, *C. nutans* has been used for treating bacterial and viral infections, scalds and burns, insect and snake bites, skin rashes, gout, and diabetes [2]. Several studies have demonstrated that it has several biological activities, including anticancer, antioxidant, antidiabetic, antiviral, anti-inflammatory, immunomodulatory, and neuromodulatory activities [1,3].

*C. nutans* has an abundance of phytochemicals [4], which involve many of its biological activities [4]. Various solvents (e.g., polar, semipolar, and nonpolar reagents) and different preparative methods have been used to retrieve these phytochemicals [5]. For example, Fazil et al. used kinetic extraction modeling to obtain the maximal yield of flavonoids from *C. nutans* water extract, and this extract displayed the best antiproliferative activity against A549 lung cancer cells [6]. However, for *C. nutans* to be applied in clinical or agricultural practice, a standardized protocol for preparing *C. nutans* extracts is required.

**Figure 1:**
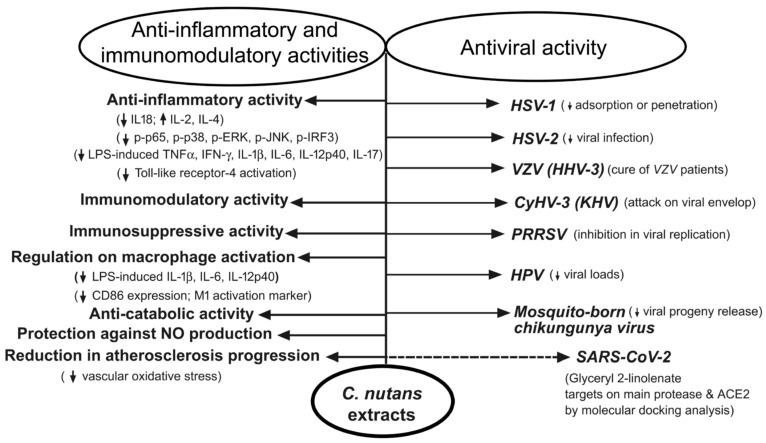
All activities of the *C. nutans* extracts that are discussed in this paper. *C. nutans* extracts display antiviral activities against herpes simplex virus type 1 (HSV-1), herpes simplex virus type 2 (HSV-2), varicella-zoster virus (VZV), cyprinid herpesvirus 3 (CyHV-3), porcine reproductive and respiratory syndrome virus (PRRSV), mosquito-borne chikungunya virus, and potentially SARS-CoV-2 through different mechanisms. In addition, phytochemicals from *C. nutans* exhibit anti-inflammatory, immunoregulatory, immunosuppressive, and anticatabolic activities; regulate macrophage activation; protect against nitric oxide (NO) production; and reduce atherosclerosis progression in type 2 diabetic rats.

In this article, we discuss all known antiviral, anti-inflammatory, and immunomodulatory activities of *C. nutans*. For this purpose, we searched PubMed and the Web of Science Core Collection databases by using the keywords “*Clinacanthus nutans* and virus”, “*Clinacanthus nutans* and inflammation”, and “*Clinacanthus nutans* and immunology” for relevant studies. For a detailed narrative review of phytochemical compounds and their health-promoting activities, please see Chia et al. [4]. This is an up-to-date review covering the antiviral, anti-inflammatory, and neuromodulatory activities of *C. nutans* extracts. All activities of the *C. nutans* extracts that are included in this study are illustrated in Figure 1.

## 2. Antiviral Activities of *C. nutans*

### 2.1. Anti-Varicella-Zoster-Virus Activity

Varicella-zoster virus (VZV), also known as human herpesvirus 3, is a common human-restricted pathogen that causes varicella or chickenpox and, subsequently, latent infection in the sensory ganglia. VZV infection leads to severe morbidity in immunocompromised hosts. Current therapy for VZV infection includes the use of antiviral agents and helicase–primase inhibitors, and a vaccine is available for primary prevention [7]. The disadvantages of using antiviral agents to treat VZV infection include low efficiency and the development of drug resistance [8]. Therefore, more effective drug treatments should be developed. Sangkitporn et al. conducted a clinical trial involving 51 patients with herpes zoster infections in which a topical formulation of *C. nutans* (*Bi Phaya Yaw*) extract was applied. Compared with a placebo, the cream containing *C. nutans* (*Bi Phaya Yaw*) extract successfully crusted and healed patients’ lesions and alleviated pain, indicating that *C. nutans* extract can be used to treat herpes zoster infections [9].

### 2.2. Anti-Herpes-Simplex-Virus Activity

Herpes simplex virus (HSV), which belongs to the alpha-herpesvirus subfamily of the Herpesviridae family, is classified into two serotypes: HSV-1 and HSV-2 [10]. Infection with HSV can cause oral or genital diseases, and severe cases may be life threatening. The infection rate increases with age [11]. Viral entry into the human host begins with an interaction between viral envelope glycoproteins and host receptors. This is followed by fusion of the envelope membrane with the host membrane [10], which is a key step in drug intervention. No effective HSV vaccines are currently available. Phytochemicals with anti-HSV activities have demonstrated promise for HSV treatment [12]. The Reutrakul group evaluated the antiviral activity of *Barleria lupulina* methanolic leaf and twig extract and *C. nutans* methanolic whole-plant extract against HSV-2 (G) and five clinical HSV-2 isolates. They observed that the *B. lupulina* extract and, to a lesser extent, *C. nutans* extract displayed strong virucidal activity, indicating that both extracts have therapeutic potential against HSV-2 [13]. Because of the antiviral activity of beta-galactosyl diglycerides in *C. nutans* leaves, a study synthesized 19 monoglycosyl diglycerides, and their anti-HSV-1 and anti-HSV-2 activities were tested. Among the compounds, 1,2-*O*-dilinolenoyl-3-*O*-beta-d-glucopyranosyl-sn-glycerol displayed the highest activities, with an IC_50_ of 12.5 ± 0.5 μg/mL recorded for HSV-1 and of 18.5 ± 1.5 μg/mL recorded for HSV-2 [14].

Three chlorophyll derivatives—132-hydroxy-(132-R)-phaeophytin b, 132-hydroxy-(132-S)-phaeophytin a, and 132-hydroxy-(132-R)-phaeophytin a—were isolated from the chloroform extracts of *C. nutans* leaves. The compounds displayed anti-HSV-1-strain-F (HSV-1F) activity, which was confirmed by a plaque reduction assay of African green monkey kidney Vero cells, with the effect likely induced by the compounds inhibiting viral adsorption or penetration [15].

Two compounds, CL03 and CL21, were purified from *C. nutans* ethanolic leaf extracts, and their anti-HSV-2 activity was evaluated using dot blotting assays of viral DNAs and Western blotting analyses of the viral proteins of HSV-2-infected Hep-2 cells. Treating HSV-2 with *C. nutans* extracts before infection substantially reduced its infectivity in Hep-2 cells and led to a depletion of viral proteins and viral structural proteins. These findings indicate that *C. nutans* leaf extracts may prevent HSV infection [16].

The authentication of herbal plants must be completed prior to drug preparation. Macroscopic, microscopic, and molecular methods can be used to authenticate the identity of *C. nutans* (Burm. f.) Lindau and *Clinacanthus siamensis* Bremek. The plants have similar anatomical structures, which led scholars to believe that they are closely related species. In addition, the plaque reduction assay was used to reveal that n-hexane, dichloromethane, and methanol leaf extracts from the two species had anti-HSV-1 (KOS) and anti-HSV-2 (Baylor 186) activities. These two *Clinacanthus* species are a rich source of phytochemicals with anti-HSV activities [17].

Monogalactosyl diglycerides (MGDG) and digalactosyl diglycerides (DGDG) isolated from the chloroform leaf extract of *C. nutans* can inhibit the late stage of replication of HSV-1 and HSV-2. A 100% inhibition of HSV-1 replication occurred at the post step of infection when MGDG and DGDG were applied at noncytotoxic concentrations to Vero cells, with MGDG and DGDG having IC_50_ values of 36.00 and 40.00 μg/mL, respectively, and of 41.00 and 43.20 μg/mL, respectively, for HSV-2. There were no antiviral activities when Vero cells were pre-treated with both chemicals before viral infection. MGDG and DGDG displayed antiviral activities against HSV-1 with selectivity indexes of 26.00 and 23.00, respectively, and HSV-2 of 23.30 and 21.30, respectively [18]. Further modifications may be required to strengthen the anti-HSV activity of MGDG and DGDG isolated from the chloroform extract of *C. nutans*.

### 2.3. Anti-Cyprinid-Herpesvirus-3 Activity in Koi Carp (Cyprinus carpio Koi)

*C. nutans* extracts display antiviral activities against a couple of viruses [19]. Cyprinid herpesvirus 3 (CyHV-3), a koi herpesvirus, causes infective disease in koi (*Cyprinus carpio koi*) and the common carp (*Cyprinus carpio* L.), which can lead to economic losses in aquaculture. *C. nutans* extract was demonstrated to possess anti-CyHV-3 activity in koi fin cells [20]. Furthermore, *C. nutans* ethanol extracts from aerial parts had anti-CyHV-3 activities in koi and increased their survival rate after viral infection [21]. *C. nutans* extract likely exerts its virucidal activity by targeting the viral envelope; the extracts worked only on enveloped viruses [22].

### 2.4. Anti-Porcine-Reproductive-and-Respiratory-Syndrome-Virus Activity

Porcine reproductive and respiratory syndrome virus (PRRSV) is an infectious agent leading to porcine reproductive and respiratory syndrome, which causes great economic losses in swine husbandry. Although vaccines against PRRSV are available, their efficacy is unsatisfactory, indicating that new strategies for disease prevention and control must be developed [23]. Arjin et al. evaluated the anti-PRRSV activities of ethanolic extracts from seven medicinal plants, namely, *Caesalpinia sappan* Linn., *Garcinia mangostana* Linn., *Houttuynia cordata*, *Perilla frutescens*, *Phyllanthus emblica*, *Tiliacora triandra*, and *C. nutans*. In this article, the MARC-145 cells, a simian cell line suitable for PRRSV propagation, was used to assay the activities of plant extracts on viral infection and replication in vivo. The antiviral screening results revealed that *T. triandra* extract was the most effective at reducing PRRSV infectivity, with the results revealing a virus titer of 3.5 TCID_50_ (log_10_) when the extract was used at a 50% cytotoxic concentration, CC_50_ (1250 μg/mL), followed by other plant extracts at their CC_50_. At higher concentrations, all plant extracts, including *C. nutans* extract, effectively inhibited virus replication in the MARC-145 cells, with the most effective being a *Cae. sappan* extract, for which the virus titer was 2.5 TCID_50_/mL (log_10_) at 72 h after infection. These activities may be due to high phenolic compounds and high antioxidant activities in these extracts [24].

### 2.5. Clearance of Condyloma Acuminata Caused by Human Papillomavirus by Using C. nutans Lindau Cream

Human papillomavirus (HPV) causes sexually transmitted infectious diseases. More than 200 types of HPV were identified, and they can be divided into high-risk or low-risk types on the basis of their oncogenicity [25]. The high-risk types are linked to malignant diseases, such as anogenital, cervical, and oropharyngeal cancers. The low-risk types are often found in condyloma acuminata (CA). A randomized clinical trial compared the effect of *C. nutans* Lindau cream with that of podophyllin, the standard agent for treating CA, on CA. The *C. nutans* cream reduced the size of CA warts and HPV viral loads by 84.4% and 46.6%, respectively, whereas podophyllin reduced the size and viral loads by 97.0% and 74%, respectively. The gene expression profile indicated that the *C. nutans* cream elicited the strong expression of 2 immune-related genes, *IFNL1* and *IRF2*, whereas podophyllin induced significant changes in 23 immune-related genes, including *HLA-DPB*, *CCL3*, *CXCL2*, *CXCR2*, and *OSM*. Further analyses of inflammation-related genes revealed that *C. nutans* cream did not induce changes in any genes, whereas podophyllin increased the expression of 108 genes. Thus, *C. nutans* treatment may activate the immune response to inhibit HPV infection, whereas podophyllin may activate the proinflammatory response [26]. In another study, the same group of researchers analyzed the treatment of patients with CA with the *C. nutans* Lindau cream or podophyllin. HPV typing of patients with CA revealed nine low-risk HPV types (6, 11, 40, 51, 62, 81, 84, CP6018, and C6108) in 9 of 10 patients, and six high-risk HPV types (16, 45, 51, 54, 58, and 59) in 4 patients. In addition, four patients had multiple infections. A median CA clearance of 82% was reported for the *C. nutans* cream, whereas a median CA clearance of 97% was reported for the podophyllin, indicating that *C. nutans* Lindau cream can serve as an alternative CA treatment [27].

### 2.6. Antiviral Activity of C. nutans Extract against the Chikungunya Virus

The chikungunya virus is an enveloped, single-stranded, positive-sense RNA virus belonging to the genus *Alphavirus* of the Togaviridae family and causes chikungunya fever [28]. More strategies for treating the disease are warranted. One study produced 132 extracts from 21 medicinal plants, including *C. nutans*, by using sequential solvent extraction and evaluated their cytopathic inhibition effect in virus-infected Vero cells by using concurrent and nonconcurrent modes. Chloroform, ethyl acetate, and ethanolic leaf extracts of *C. nutans* produced through the concurrent mode significantly inhibited the chikungunya virus’ cytopathic effects and viral progeny release, with the extracts having selectivity index values of 4.99, 13.39, and >20.45, respectively. These *C. nutans* extracts likely suppressed chikungunya virus activities by inhibiting viral progeny release [29].

### 2.7. Potential Anti-SARS-CoV-2 Activity

The COVID-19 pandemic, which was caused by SARS-CoV-2, led to the death of millions of people [30]. In addition to vaccines and drugs, phytochemicals may help in treating SARS-CoV-2 [31]. The main protease of SARS-CoV-2 (3CL^pro^), which is critical for viral replication, may serve as a target for phytochemical treatment [31]. In molecular docking analyses, nine compounds were revealed to potentially inhibit the SARS-CoV-2 main protease: (3,3-dimethylally) isoflavone, licoleafol, myricitrin, thymoquinone, salvinorin A, bilobalide, citral, ginkgolide A, and perphenazine [32]. Tannic acid, which is present in many fruits and vegetables, can also target the SARS-CoV-2 main protease [33,34]. These compounds could have therapeutic applications and lessen the severity of SARS-CoV-2 infection in the future.

Phytochemicals from *C. nutans* extracts may also aid in COVID-19 therapy [35]. A study detected compounds in *C. nutans* dichloromethane extract obtained from fresh leaves by using gas chromatography mass spectrometry, and 14 compounds with similarity indexes of 80–99% [35] were isolated; palmitic acid and linolenyl alcohol were the most abundant. Drug-likeness and toxicity analyses indicated that all of the compounds can be used as oral drugs, with some compounds being mutagenic, tumorigenic, and toxic to some organs. Molecular docking analyses revealed that one of the compounds, glyceryl 2-linolenate, exhibited the highest binding affinity toward both the SARS-CoV-2 main protease and ACE2 receptor proteins, indicating that this compound (or a further-modified version of it), can be used clinically to manage SARS-CoV-2 infection [35]. Compounds isolated from other extraction methods using different solvents also can be applied with this same strategy to look for more effective anti-SARS-CoV-2 phytochemicals.

## 3. Anti-Inflammatory and Immunomodulatory Activities of *C. nutans*

### 3.1. Suppression of Neutrophil-Dependent Inflammation by C. nutans Extract

Whole-plant methanolic *C. nutans* extract significantly and dose-dependently inhibited carrageenan-induced paw edema and ethyl-phenylpropiolate-induced ear edema in rats. *C. nutans* extract suppressed neutrophil functional responsiveness by inhibiting *N*-formyl-methionyl-leucyl-phenylalanine (fMLP)-induced chemotaxis, superoxide anion generation, myeloperoxidase, and elastase release. Therefore, *C. nutans* extract displays strong anti-inflammatory activities because it partially inhibits neutrophil responsiveness [36].

### 3.2. In Vivo Immunomodulatory Activity of C. nutans 30% Ethanol Extract in HepA Xenograft Mice

Hepatocellular carcinoma (HCC) accounts for 90% of liver cancers and is the fourth leading cause of cancer-related deaths worldwide [37]. Researchers estimated that by 2030, HCC will be the cause of approximately 1 million deaths annually [38]. Current drugs for HCC treatment, including fluorouracil, Cytoxan, cisplatin, doxorubicin, and vincristine, have nonspecific cytotoxicities and adverse effects, indicating that novel agents should be developed. *C. nutans* 30% ethanol extract (CN30) containing seven major components (shaftoside, orientin, vitexin, isoorientin, isovitexin, 6,8-apigenin-*C*-α-L-pyranarabinoside, and gallic acid) was observed to inhibit tumor growth in HepA xenograft mice through inducing apoptosis. CN30 displayed immunomodulatory activity, and CN30 treatment increased the serum interferon (IFN)-γ and interleukin (IL)-2 levels in tumor-bearing mice. It also raised the ratio of IFN-γ^+^ CD4^+^ T cells (Th1), slightly decreased the level of IL-4^+^ CD4^+^ T cells (Th2), and did not change the levels of IL-17A^+^ CD4^+^ T cells and FOXP3^+^ CD4^+^ T cells in treated tumor-bearing mice. Together, these findings indicate that CN30 was effective at treating HCC in a mouse model because of its antitumor and immunomodulatory activities [39].

### 3.3. Anti-Inflammatory Effects of C. nutans Extracts on the Inhibition of Cytokine Production and Toll-like Receptor-4 Activation

Four *C. nutans* extracts—polar leaf extract (LP), nonpolar leaf extract (LN), polar stem extract (SP), and nonpolar stem extract (SN)—were prepared, and their anti-inflammatory effects were tested by assaying the LPS-induced nitrite release in RAW264.7 macrophages and Toll-like receptor (TLR-4) activation in TLR-4-transfected human embryonic kidney cells (HEK-Blue^TM^-hTLR4 cells). All four extracts were nontoxic to RAW264.7 cells and HEK-Blue^TM^-hTLR4 cells, as evidenced by MTT assay and morphology examination results. All four extracts reduced the amount of LPS-induced nitrite release and LPS-induced secretion of inflammatory cytokines (TNF-α, IFN-γ, IL-1β, IL-6, IL-12p40, and IL-17) in RAW264.7 macrophages as well as suppressed LPS-induced TLR-4 activation in HEK-Blue^TM^-hTLR4 cells, with LP being the most effective extract. LP treatment also reduced the LPS-induced active form of inflammatory proteins, including phosphorylated p65, p38, ERK, JNK, and IRF3. The anti-inflammatory activities of LP extract could be due to its high content of total phenolic compounds [40].

### 3.4. Phytosterols Isolated from C. nutans with Immunosuppressive Activity

Phytosterols, the sterols isolated from plants, display multiple biological activities, including anti-inflammatory, antioxidant, anticancer, antidiabetic, antiatherosclerotic, antieryptotic, antihemolytic, neuroprotective, and microbiota-modulatory activities [41]. A study isolated four phytosterols, namely, shaftoside (CN1), stigmasterol (CN2), β-sitosterol (CN3), and a triterpenoid lupeol (CN4), from the hexane extracts of *C. nutans* leaves, and their immunosuppressive activities were analyzed. CN2 and CN3 inhibited concanavalin A (ConA)-induced T cell proliferation, whereas only CN3 blocked the secretion of T helper 2 (Th2) cytokines, including IL-4 and IL-10. However, neither affected the secretion of Th1 cytokines, including IL-2 and IFN-γ. CN3 also decreased the proliferation of CD4^+^CD25^+^ T helper cells and CD8^+^CD25^+^ cytotoxic T cells after the challenge of T cells with ConA. The study indicated that hexane extracts of *C. nutans* leaves have immunosuppressive activity [42].

### 3.5. Evaluation of the Protective Effect of C. nutans Water Extract against Nitric Oxide Production in LPS-IFN-γ-Activated RAW 264.7 Macrophages by ^1^H-NMR Metabolomics

Five types of solvents (100%, 70%, 50%, and 20% ethanol and 100% water) and two extraction methods (soaking and ultrasound-assisted extraction) were used to obtain *C. nutans* extracts from dry leaves, and their anti-inflammatory activity was evaluated by analyzing the nitric oxide (NO) inhibition effect in lipopolysaccharide (LPS)-IFN-γ-activated RAW 264.7 macrophages. The sonicated water extract displayed the highest NO inhibition activity (IC_50_ = 190.43 ± 12.26 μg/mL, *p* < 0.05). ^1^H-nuclear magnetic resonance (NMR) metabolomic analysis indicated that 56 compounds were involved. A partial least squares biplot analysis revealed the following compounds to be potential NO inhibitors: sulfur-containing glucoside, sulfur-containing compounds, phytosterols, triterpenoids, flavones, and some organic and amino acids. Furthermore, liquid chromatography tandem mass spectrometry revealed that sonicated water extract possesses the highest abundance of C-glycosyl flavones [43]. This finding not only revealed that *C. nutans* components can inhibit NO activity in LPS-IFN-γ-activated RAW 264.7 macrophages, but also revealed a method through which phytochemicals and their link to bioactivity can be identified. This finding can be used to improve the speed of effective component identification and reveals potential clinical applications for *C. nutans* extracts.

### 3.6. Anti-Inflammatory Effects of C. nutans Leaf Extract on the Brain

A pioneer study of the anti-inflammatory effects of *C. nutans* leaf extract on the brain was initiated by Ahmad Azam et al. LPS-injected rats were fed *C. nutans* aqueous leaf extract (CNE; 1000 and 500 mg/kg body weight [BW]) or the positive control drug dextromethorphan (5 mg/kg BW) for 14 days. Subsequently, ^1^H-NMR and cytokine microarray analyses were conducted on the brain tissues of the treated group. Principal component analysis of the NMR spectral data revealed 21 metabolites in the brain tissue to be biomarkers of LPS-induced neuroinflammation. Compared with a negative control treatment, CNE at 1000 and 500 mg/kg BW led to a change in the amounts of some metabolites, including lactate, pyruvate, phosphorylcholine, glutamine, and α-ketoglutarate. Similar to treatment with dextromethorphan, treatment with CNE displayed antineuroinflammatory potential, as revealed by statistical isolinear multiple component analysis of NMR spectral data. According to the results of a cytokine microarray analysis, both 1000 and 500 mg/kg BW CNE led to a greater decrease in the proinflammatory cytokine (IL-1β) than dextromethorphan. CNE treatment also upregulated the anti-inflammatory cytokines IL-2 and IL-4. These data indicate that CNE can effectively suppress neuroinflammation [44]. In addition, they indicate that metabolomics can be used to determine the effects of CNE on the metabolism of affected tissues.

### 3.7. Anti-Inflammatory and Anticatabolic Activities of Apigenin-C-Glycoside-Rich C. nutans Leaf Aqueous Ethanol Extract in an Osteoporotic/Osteoarthritis Rat Model

Diclofenac is a nonsteroidal anti-inflammatory drug used to control inflammation-related symptoms of osteoarthritis and osteoporosis, which usually occur in older people. However, its prolonged use results in adverse effects, such as gastrointestinal and cardiovascular problems [45] and impaired fracture healing [46]. Phytochemicals that reduce inflammation may be another option for controlling the symptoms of osteoarthritis and osteoporosis. Therefore, a study investigated the anti-inflammatory and anticatabolic activities of *C. nutans* leaf aqueous ethanol extract (CNAE), which is rich in apigenin C-glycoside, in osteoporotic/osteoarthritic rats. High performance liquid chromatography (HPLC) analyses indicated that the CNAE had an abundance of apigenin C-glycosides, including shaftoside (apigenin 8-C-glucoside-6-C-arabinoside), vitexin (apigenin 8-C-glucoside), and isovitexin (apigenin 6-C-glucoside), and a small amount of isoschaftoside (apigenin 6-C-arabinoside-8-C-glucoside). The quantities of apigenin-C-glycosides present when 400 mg CNAE/kg (equivalent to 0.2 mg apigenin equivalent/kg) and the positive control drug diclofenac (5 mg/kg) were administered were sufficient to reduce bone loss, cartilage erosion, and cartilage catabolic changes in the osteoporotic/osteoarthritic rats. CNAE treatment also led to a decrease in the amount of the proinflammatory cytokine interleukin-1-beta (IL-1β) and the osteoarthritis marker procollagen type II N-terminal propeptide, and to an increase in the osteoporosis markers procollagen type I N-terminal propeptide (PINP) and osteocalcin in the serum of the osteoporotic/osteoarthritic rats that were comparable to those that occurred when diclofenac treatment was administered. In addition, CNAE treatment led to a reduction in the mRNA expression in proinflammatory markers and proteolytic enzymes, including IL-1β, nuclear-factor-kappa-beta (NF-κβ), cyclooxygenase-2 (COX-2), and matrix-metalloproteinase-13, in the osteoporotic/osteoarthritic rats that was comparable to those induced by diclofenac. These findings indicate that the apigenin-C-glycosides-rich *C. nutans* leaf extract can reduce inflammation and protect against bone loss and cartilage destruction in osteoporotic/osteoarthritic rats by suppressing the inflammatory and catabolic protease pathways with an efficacy comparable to that of diclofenac [47].

### 3.8. Protection against 7-Ketocholesterol-Induced Damage and Inflammation in Human Cerebral Microvascular Endothelial Cells

Oxosterol 7-ketocholesterol (7KC) is present in the blood of patients with diabetes mellitus [48] and cardiovascular disease [49,50]. Treatment of brain endothelial cells with 7KC resulted in the induction of reactive oxygen species, apoptosis, inhibition of cell cycle progression, and increased secretion of the inflammatory cytokine IL-8 [51]. hCMEC/D3 cells, a human cerebral microvascular endothelial cell line that originates from human temporal lobe microvessels, are reported to possess typical brain endothelial cell properties [52]. 7KC reduces the cell viability of -hCMEC/D3 cells dose-dependently. However, *C. nutans* methanolic leaf extract (but not *C. nutans* methanolic stem extract) was reported to reverse this activity. 7KC-induced elevation of the expression of the proinflammatory cytokines IL-8, IL-6, IL-1β, TNF-α, and COX-2 was reduced when *C. nutans* methanolic leaf extract, instead of stem extract, was used. HPLC analysis revealed that completely different components were present in *C. nutans* leaf and stem extracts. This finding supports the notion that *C. nutans* leaf extract has unique effects. *C. nutans* methanolic leaf extract has potential for treating cardiovascular disease and diabetes mellitus [53].

### 3.9. Immunomodulatory Activities of C. nutans Ethanol and Water Extracts in the Coculture of Triple-Negative Breast Cancer MDA-MB-231 Cells and Differentiated thp-1 Macrophages

Triple-negative breast cancer (TNBC), which accounts for 15–20% of all breast cancers [54], does not have estrogen, progesterone, and epidermal growth-factor-2 receptors and is therefore challenging to manage. The tumor microenvironment (TME) plays a critical role in tumor progression [55]. By targeting the TME, especially immune cells, we may have chance to turn the immune system into an anti-cancer weapon for the treatment of TNBC. A study used *C. nutans* ethanol and water extracts to treat a coculture of TNBC MDA-MB-231 cells and differentiated THP-1 macrophages to complete an assay of their effects on chronic inflammation in the TME. Neither extract was toxic to MDA-MB-231 cells or THP-1 cells, and neither extract affected the migration of MDA-MB-231 cells. Compared with LPS-induced control cells, *C. nutans* ethanol extract reduced IL-6 levels at 25 μg/mL and 100 μg/mL, whereas *C. nutans* water extract increased IL-6 levels at 50 μg/mL and 100 μg/mL. Both extracts reduced IL-1β levels at 25 μg/mL. *C. nutans* ethanol extract substantially reduced TNF-α levels, whereas the water extract slightly inhibited TNF-α levels at all extract concentrations. These results indicate that *C. nutans* extracts have anti-inflammatory and immunomodulatory activities in the TME of TNBC [56].

### 3.10. C. nutans Leaf Methanol Extract Reduces Atherosclerosis Progression in Type 2 Diabetic Rats by Reducing Vascular Oxidative Stress and Inflammation

Diabetes mellitus is a risk factor for atherosclerosis and cardiovascular disease [57]. Atherosclerotic cardiovascular disease is the main cause of death in type 2 diabetes (T2DM), which is believed to be a proinflammatory condition. Therefore, anti-inflammatory strategies should be employed for patients with T2DM [58]. Because *C. nutans* extract has anti-inflammatory activities [40], a study investigated the antiatherosclerotic activity of *C. nutans* leaf methanol extract (CNME) in a T2DM rat model, with T2DM induced by a high-fat diet and low-dose streptozotocin. CNME treatment reduced the rats’ blood cholesterol, triglycerides, low-density lipoprotein cholesterol, atherogenic index values, malondialdehyde levels, TNF-α levels, and intima–media thicknesses to an extent comparable to that of metformin, but increased their superoxide dismutase levels. These findings confirm that CNME’s anti-inflammatory and antioxidant activities can ameliorate atherosclerotic symptoms in T2DM [59].

### 3.11. C. nutans Extracts Regulate the Macrophage Cellular Response

Macrophage is constitutively activated in certain inflammatory diseases, but its activation is absent in certain macrophage-related immunodeficiency disorders, demonstrating the importance of regulation in macrophage activation. A study analyzed the effects of *C. nutans* ethanol, ethanol–aqueous, and aqueous leaf extracts on the regulation of the macrophage cellular response in mouse J774.2 macrophages. None of the extracts at any tested concentrations were significantly cytotoxic. Lipopolysaccharide-induced proinflammatory cytokines, including IL-1β, IL-6, and IL-12p40, and the expression of the M1 activation marker CD86, were downregulated by a 50% ethanol–aqueous extract, whereas the phagocytic activity of J774.2 macrophages was augmented. These data indicate that *C. nutans* extracts may be useful for treating macrophage-activation-related diseases [60].

## 4. Concluding Remarks and Future Directions

*C. nutans* is rich in phytochemicals [5], which are responsible for its health-promoting activities [5], including its antiviral, anti-inflammatory, and immunomodulatory activities. Phytochemicals isolated from different plant parts using different solvents display differential activities against various viruses (Table 1). *C. nutans* methanolic leaf extracts display anti-HSV-1 (KOS) and anti-HSV-2 (Baylor 186) activities [17], whereas *C. nutans* ethanolic extracts from aerial parts exhibit virucidal activity against CyHV-3 [21] and those from leaves exhibit anti-chikungunya-virus activity by targeting virus progeny release [29]. Three chlorophyll derivatives isolated from *C. nutans* chloroform leaf extract were reported to possess anti-HSV-1-strain-F activity that probably resulted from their interference with virus infection or penetration [15]. Compounds from *C. nutans* dichloromethane leaf extracts may be effective against SARS-CoV-2 infection because they may target the main protease (3CL^pro^) and ACE2 receptors of SARS-CoV-2 based on the molecular docking analyses [35]; this topic warrants further research. Continued study on the direct interaction of the selected chemical with the main protease of the SARS-CoV-2 virus and ACE2 receptor could be detected by using Affi-gel affinity chromatography [61] or surface plasmon resonance screening [62]. Bioassays of selected chemicals on thereplication of the SARS-CoV-2 virus by interfering with the activity of the main protease, and on viral infection by interrupting the interaction of the SARS-CoV-2 virus with the ACE2 receptor, could also be studied. Together, these studies indicate that phytochemicals from *C. nutans* can act against various viruses at multiple stages of viral infection.

The anti-inflammatory and immunomodulatory activities of *C. nutans* extracts are outlined in Table 2. Among these studies, the discovery of CNE in alleviating the neuroinflammation is impressive [44]. As drugs do not easily cross the blood–brain barrier (BBB), which lessens the drug’s effect [63], this activity of *C. nutans* extract on the brain is important. How *C. nutans* extract can cross the BBB to produce drug effects and its underlying molecular mechanism of anti-neuroinflammation are worthy of continued study. Ethanol and methanol are two solvents commonly used to extract phytochemicals associated with anti-inflammatory and immunoregulatory activities. CN30, whose major components include shaftoside, orientin, vitexin, isoorientin, isovitexin, 6,8-apigenin-*C*-α-L-pyranarabinoside, and gallic acid, upregulated the immune response to inhibit tumor growth in HepA xenograft model mice [39]. *C. nutans* leaf aqueous ethanol extract, which is rich in apigenin-C-glycosides, was able to lessen inflammation, bone loss, and cartilage destruction in osteoporotic/osteoarthritic rats by inhibiting the inflammatory and catabolic proteases pathways [47]. Another report indicated that *C. nutans* ethanol and *C. nutans* water extracts both displayed anti-inflammatory and immunomodulatory activities in the TME of TNBC cells [56]. Another study reported that *C. nutans* methanolic extracts could inhibit inflammation in a diabetic animal model [59]. Regarding water solvents, CNE displayed antineuroinflammatory activity [44], and sonicated water extract displayed the highest NO inhibition activity [43]. *C. nutans* hexane leaf extracts that are rich in phytosterols have immunosuppressive activity [42]. Regardless of whether extracts have been isolated using ethanol, methanol, water, hexane, or other solvents, the phytochemicals in the extracts have consistently displayed anti-inflammatory and immunoregulatory activities.

Many studies have delineated the activities of *C. nutans* extracts against virus, inflammation, and on immunomodulation; however, a systemic analysis of these effects is lacking. Systemic approaches such as metabolomics, proteomics, RNA sequencing, and combined analysis with bioinformatics could be employed to have a full picture of dynamic fluctuation in the levels of genes, proteins of signaling pathways, or metabolites in metabolic pathways during treatment with *C. nutans* extracts, and thus providing information for the construction of fine and integrated networks of drug effects on antiviral, anti-inflammation, and immunomodulation for a better understanding of the action mechanisms of *C. nutans* extract and more effective treatment of related diseases.

Although *C. nutans* possesses various health-promoting activities, including antiviral, anti-inflammatory, and immunomodulatory activities, the toxicity of *C. nutans* extracts should be carefully evaluated when it is used for treatment. A study of the *C. nutans* polar methanol leaf extract on Sprague Dawley (SD) rats showed that the extract was considered safe at all tested dosages of 0.3 g/kg, 0.6 g/kg, and 0.9 g/kg of BW up to 14 days. No adverse effects and obvious organ damage were observed in SD rats [64]. In an acute oral toxicity assay of mice, no adverse effects were observed when the *C. nutans* methanol leaf extract of more than 2500 mg/kg BW was applied, with the LD_50_ of *C. nutans* extract of more than 5000 mg/kg BW [65]. A toxicity assay of methanol leaf extract on 4T1 tumor-bearing mice showed no adverse effects and inflammatory responses at doses as high as 1000 mg/kg BW [66]. These studies indicate that *C. nutans* extract appears to be nontoxic with no obvious side effects. However, the extract might not be suitable for fragile patients. The benefits of *C. nutans* extract could outweigh its side effects, if suitable dosages are applied.

Optimizing solvents and extraction methods for preparing *C. nutans* extracts is crucial for treating various diseases because the type and yield of phytochemicals vary with the extraction method and solvent polarity [5]. In addition, in *C. nutans* extracts, active compounds can be identified alone or in combination through functional bioassay and mass spectrometry. After cellular targets have been identified, active compounds can be modified to be more effective against viruses, inflammation, and on immunomodulation through molecular docking [67,68] or medicinal chemistry [69,70]. The synthetic biology and CRISPR technology have been successfully used in metabolic engineering of key enzymes and regulators into medicinal plants to enhance the content of alkaloids in plants [71]. The same strategy can be applied to *C. nutans* to increase the levels of the active components for disease treatment. The current review can advance the understanding of the antiviral, anti-inflammatory, and immunomodulatory activities of *C. nutans* extracts, and the results of this study can be used to identify agricultural and clinical applications of such extracts.

## Figures and Tables

**Table 1:** Antiviral activities of *Clinacanthus nutans* extracts.

Type of Virus	Type of Extract and Source Plant Part(s)	Biological Activity	Reference
Varicella-zoster virus (VZV)	Cream of *Clinacanthus nutans (Bi Phaya Yaw)* extract	Cream was successful in crusting and healing the lesions caused by herpes zoster infection.	Sangkitporn et al., 1995 [9]
Herpes simplex virus type 2 strain G [HSV-2 (G)] and five clinical herpes simplex virus type 2 (HSV-2) isolates	Whole-plant *C. nutans* methanolic extract	*C. nutans* extract displayed virucidal activities.	Yoosook et al., 1999 [13]
Herpes simplex virus type 1 (HSV-1) and HSV-2	Synthesized 19 monoglycosyl diglycerides	Out of 19 compounds, 1,2-*O*-dilinolenoyl-3-*O*-beta-d-glucopyranosyl-sn-glycerol displayed the highest anti-HSV-1 and anti-HSV-2 activities, with an IC_50_ of 12.5 ± 0.5 μg/mL noted for HSV-1 and of 18.5 ± 1.5 μg/mL noted for HSV-2.	Janwitayanuchit et al., 2003 [14]
HSV-1 strain F (HSV-1F)	Chloroform extracts of *C. nutans* leaves; three chlorophyll derivatives, namely, 132-hydroxy-(132-R)-phaeophytin b, 132-hydroxy-(132-S)-phaeophytin a, and 132-hydroxy-(132-R)-phaeophytin a, were isolated	Compounds displayed antiviral activity possibly resulting from their inhibition of viral adsorption or penetration.	Sakdarat et al., 2009 [15]
HSV-2	Two purified compounds, CL03 and CL21, obtained from *C. nutans* ethanolic leaf extracts	Treatment of the virus with the extracts before infection substantially reduced the viral infectivity. Depletion or decrease in viral proteins and viral structural proteins was observed.	Vachirayonstien et al., 2010 [16]
HSV-1 and HSV-2	N-hexane, dichloromethane, and methanol leaf extracts from two species, *C. nutans* (Burm. f.) Lindau (*C. nutans*) and *Clinacanthus siamensis* Bremek (*C. siamensis*)	All leaf extracts from the two species displayed anti-HSV-1 (KOS) and anti-HSV-2 (Baylor 186) activities.	Kunsorn et al., 2013 [17]
HSV-1 and HSV-2	Monogalactosyl diglycerides (MGDG) and digalactosyl diglycerides (DGDG) isolated from chloroform extract of *C. nutans*	MGDG and DGDG displayed anti-HSV-1 and anti-HSV-2 activities at the late stage of multiplication. MGDG and DGDG exhibited 100% inhibition of HSV-1 and HSV-2 replication at the post step of infection at noncytotoxic concentrations with IC_50_ values of 36.00 and 40.00 μg/mL and of 41.00 and 43.20 μg/mL, respectively. MGDG and DGDG exhibited antiviral activities against HSV-1 and HSV-2 with selectivity indexes of 26.00 and 23.00 and of 23.30 and 21.30, respectively.	Pongmuangmul et al., 2016 [18]
Cyprinid herpesvirus 3 (CyHV-3)	*C. nutans* ethanol extracts from aerial parts	Extract had anti-CyHV-3 activities and increased the survival rates of fishes after viral infection.	Haetrakul et al., 2018 [20]
Porcine reproductive and respiratory syndrome virus (PRRSV)	Ethanolic extracts from seven medicinal plants, namely, *Caesalpinia sappan* Linn., *Garcinia mangostana* Linn., *Houttuynia cordata*, *Perilla frutescens*, *Phyllanthus emblica*, *Tiliacora triandra*, and *C. nutans*.	All plant extracts, including *C. nutans,* effectively inhibited PRRSV replication at higher concentrations.	Arjin et al., 2020 [24]
Human papillomavirus (HPV)	*C. nutans* cream (Government Pharmaceutical Organization of Thailand) or podophyllin.	*C. nutans* cream efficiently reduced the size of condyloma acuminata (CA) warts and HPV viral loads. *C. nutans* cream elicited strong expression of two immune-related genes, *IFNL1* and *IRF2*, whereas podophyllin induced significant changes in 23 immune-related genes, including *HLA-DPB*, *CCL3*, *CXCL2*, *CXCR2*, and *OSM*. *C. nutans* cream treatment did not induce changes in any inflammation-related genes, whereas podophyllin increased the expression of 108 genes. *C. nutans* extract treatment activated the immune response to inhibit HPV infection, whereas podophyllin might have activated the proinflammatory response to suppress viral infection.	Jantaravinid et al., 2021 [26]
	*C. nutans* Lindau cream (4.343 g *C. nutans* extract powder in 100 g cream) in addition to podophyllin.	Successful clearance of CA caused by HPV with an 82% clearance rate for *C. nutans* Lindau cream. Most HPV types had a low risk (HPV 6 and 11). *C. nutans* may be beneficial as an adjuvant in CA treatment.	Jiamton et al., 2022 [27]
Mosquito-born chikungunya virus	132 extracts obtained through sequential solvent extraction from 21 medicinal plants, including *C. nutans*	Chloroform, ethyl acetate, and ethanolic extracts of *C. nutans* by the concurrent mode exhibited a significant cytopathic effect and inhibitory activity. Chloroform, ethyl acetate, and ethanolic extracts of *C. nutans* inhibited viral progeny release from virus-infected cells.	Chan et al., 2021 [29]
Severe acute respiratory syndrome coronavirus 2 (SARS-CoV-2)	*C. nutans* dichloromethane extract compounds obtained from fresh leaves	Fourteen compounds from the dichloromethane extract displayed similarity indexes of 80–99%. Drug-likeness and toxicity analyses revealed that all compounds could potentially be used as oral drugs. Glyceryl 2-linolenate exhibited the highest binding affinity toward both SARS-CoV-2 main protease and ACE2 receptor proteins, indicating it may protect against SARS-CoV-2 infection.	Ismail et al., 2022 [35]

**Table 2:** Anti-inflammatory and immunomodulatory activities of *C. nutans* extracts.

Type of Extract and Source Plant Part(s)	Biological Activities	Reference
*C. nutans* methanolic extract of whole plants	*C. nutans* methanolic extract significantly inhibited carrageenan-induced paw edema and ethyl-phenylpropiolate-induced ear edema in rats. *C. nutans* extract displayed strong anti-inflammatory activities resulting from it partially inhibiting neutrophil responsiveness.	Wanikiat et al., 2008 [36]
*C. nutans* 30% ethanol extract (CN30) of aerial parts	CN30 contains seven major components, namely, shaftoside, orientin, vitexin, isoorientin, isovitexin, 6,8-apigenin-*C*-α-L-pyranarabinoside, and gallic acid. CN30 inhibited tumor growth in HepA xenograft mice by inducing apoptosis. CN30 displayed immunomodulatory activity: (1) CN30 treatment increased the serum IFN-γ and IL-2 levels in tumor-bearing mice. (2) CN30 raised the ratio of IFN-γ^+^ CD4^+^ T cells (Th1), slightly decreased the level of IL-4^+^ CD4^+^ T cells (Th2), and did not change the levels of IL-17A^+^ CD4^+^ T cells and FOXP3^+^ CD4^+^ T cells in treated tumor-bearing mice. CN30 exhibited both antitumor and immunomodulatory activities.	Huang et al., 2015 [39]
Four *C. nutans* extracts, namely, polar leaf extract (LP), nonpolar leaf extract (LN), polar stem extract (SP), and nonpolar stem extract (SN)	All four extracts were nontoxic to RAW264.7 cells and HEK-Blue-hTLR4 cells. Treatment with each extract decreased LPS-induced nitrite release and LPS-induced secretion of inflammatory cytokines (TNF-α, IFN-γ, IL-1β, IL-6, IL-12p40, and IL-17) in RAW264.7 macrophages. Treatment with each extract suppressed LPS-induced TLR-4 activation in HEK-Blue-hTLR4 cells, with LP being the most effective. Treatment with LP reduced the LPS-induced active form of inflammatory proteins, including phosphorylated p65, p38, ERK, JNK, and IRF3. LP’s anti-inflammatory activities may be the result of its high content of total phenolic compounds.	Mai et al., 2016 [40]
Hexane extracts of *C. nutans* leaves	Four phytosterols, namely, shaftoside (CN1), stigmasterol (CN2), β-sitosterol (CN3), and a triterpenoid lupeol (CN4), were isolated from the hexane extracts of *C. nutans* leaves, and their immunosuppressive activities were tested. CN2 and CN3 inhibited concanavalin-A-induced T cell proliferation, whereas only CN3 blocked the secretion of T helper 2 (Th2) cytokines, including IL-4 and IL-10. Neither CN2 nor CN3 affected the secretion of Th1 cytokines, including IL-2 and IFN-γ. CN3 reduced the proliferation of both CD4^+^CD25^+^ T helper cells and CD8^+^CD25^+^cytotoxic T cells after the challenge of T cells with concanavalin A. Hexane extracts of *C. nutans* leaves have immunosuppressive activity.	Le et al., 2017 [42]
*C. nutans* extracts obtained from dry leaves through soaking and ultrasound-assisted extraction and by using five solvents (100%, 70%, 50%, and 20% ethanol and 100% water)	Sonicated water extract displayed the highest nitric oxide (NO) inhibition activity (IC_50_ = 190.43 ± 12.26 μg/mL). ^1^H-nuclear magnetic resonance (NMR) metabolomics analyses indicated that 56 compounds were involved. Potential NO inhibitors, including sulfur-containing glucoside, sulfur-containing compounds, phytosterols, triterpenoids, flavones, and some organic and amino acids, were identified using partial least squares biplot analysis. Sonicated water extract had the highest abundance of C-glycosyl flavones.	Khoo et al., 2019 [43]
*C. nutans* aqueous leaf extract (CNE)	Principal component analysis of NMR spectral data after CNE intervention revealed 21 metabolites in brain tissue as biomarkers of LPS-induced neuroinflammation. CNE at 1000 and 500 mg/kg body weight (BW) changed the content of some metabolites, including lactate, pyruvate, phosphorylcholine, glutamine, and α-ketoglutarate, compared with that in a negative control. Treatment with CNE and the positive control drug dextromethorphan exhibited antineuroinflammatory potential, as revealed by statistical isolinear multiple component analysis. CNE at 1000 and 500 mg/kg BW reduced the content of proinflammatory cytokines IL-1β, indicating it had an effect superior to that of dextromethorphan. CNE treatments upregulated the anti-inflammatory cytokines IL-2 and IL-4.	Ahmad Azam et al., 2020 [44]
Apigenin-C-glycosides-rich *C. nutans* leaf aqueous ethanol extract (CN extract)	Treatment with apigenin-C-glycosides (400 mg CN extract/kg) and diclofenac (5 mg/kg) reduced bone loss, cartilage erosion, and cartilage catabolic changes in osteoporotic/osteoarthritic rats. Compared with treatment with diclofenac, treatment with CN extract reduced the proinflammatory cytokine interleukin-1-beta (IL-1β) and osteoarthritis marker procollagen type II N-terminal propeptide concentrations and increased the osteoporosis markers procollagen type I N-terminal propeptide (PINP) and osteocalcin concentrations in the serum of osteoporotic/osteoarthritic rats. CN extract reduced the mRNA expression of proinflammatory markers and proteolytic enzymes, including IL-1β, nuclear-factor-kappa-beta (NF-κβ), cyclooxygenase-2, and matrix-metalloproteinase-13 in osteoporotic/osteoarthritic rats. Apigenin-C-glycoside-rich *C. nutans* leaf extract reduced inflammation and protected against bone loss and cartilage destruction in osteoporotic/osteoarthritic rats by inhibiting the inflammatory and catabolic protease pathways, with an efficacy comparable to that of diclofenac.	Tantowi et al., 2020 [47]
*C. nutans* methanolic leaf extract and stem extract	7KC decreased the cell viability of human hCMEC/D3 cells, and unlike *C. nutans* methanolic stem extract, *C. nutans* methanolic leaf extract reversed this activity. *C. nutans* methanolic leaf extract decreased 7-KC-induced elevations in the expression of the proinflammatory cytokines IL-8, IL-6, IL-1β, TNF-α, and cyclooxygenase-2. *C. nutans* methanolic leaf extract displayed anti-inflammatory activity.	Kuo et al., 2021 [53]
*C. nutans* (CN) ethanol and water extracts	Neither CN ethanol extract nor CN water extract was toxic to MDA-MB-231 cells and THP-1 cells, and they did not affect the migration of MDA-MB-231 cells. Both 25 and 100 μg/mL CN ethanol extract reduced IL-6 levels, and 50 and 100 μg/mL CN water extract increased IL-6 levels relative to those when lipopolysaccharide (LPS)-induced control cells were used. Both extracts reduced IL-1β levels when applied at a concentration of 25 μg/mL. CN ethanol extract substantially reduced TNF-α levels, whereas CN water extract slightly inhibited TNF-α levels at all concentrations. CN extracts demonstrate anti-inflammatory and immunomodulatory activities in the tumor microenvironment of triple-negative breast cancer.	Nordin et al., 2021 [56]
*C. nutans* leaf methanol extract (CNME)	CNME treatment reduced blood cholesterol, triglycerides, low-density lipoprotein cholesterol, atherogenic index, malondialdehyde, TNF-α, and intima–media thickness to an extent comparable with that of the drug metformin, which is frequently prescribed to lower blood sugar levels, but increased superoxide dismutase levels in diabetic rats.	Azemi et al., 2021 [59]
*C. nutans* ethanol, 50% ethanol–aqueous, and aqueous leaf extracts	50% ethanol–aqueous extract downregulated lipopolysaccharide-induced proinflammatory cytokines, including IL-1β, IL-6, and IL-12p40, and the expression of the M1 activation marker CD86. 50% ethanol–aqueous extract increased the phagocytic activity of J774.2 macrophages.	Mohamed et al., 2022 [60]

## Data Availability

Data sharing is not applicable to this article.
